# Access to healthcare for street sex workers in the UK: perspectives and best practice guidance from a national cross-sectional survey of frontline workers

**DOI:** 10.1186/s12913-022-07581-7

**Published:** 2022-02-11

**Authors:** Lucy C. Potter, Jeremy Horwood, Gene Feder

**Affiliations:** Centre for Academic Primary Care, Department of Population Health Sciences, Bristol Medical School, University of Bristol, Bristol, UK

**Keywords:** Access to health care, Sex work, Inequalities, Inclusion health, Best practice

## Abstract

**Background:**

Street sex workers (SSWs) are a highly marginalised and stigmatised group who carry an extremely high burden of unmet health need. They experience multiple and interdependent health and social problems and extreme health inequality. Despite high levels of chronic physical and mental ill-health, there is little evidence of effective healthcare provision for this group. They are often considered ‘hard to reach’, but many individuals and organisations have extensive experience of working with this group.

**Methods:**

We conducted a cross-sectional survey of professionals who work with SSWs in the UK on their perspectives on their access to primary care, mental health, sexual health and drug and alcohol services, how well these services met the needs of SSWs and suggestions of best practice.

**Results:**

50 professionals mostly from England, responded. Mainstream general practice and mental health services were found to be largely inaccessible to SSWs. Sexual health, drug and alcohol services and homeless health services better met their needs; this was mostly attributed to flexible services and collaborations with organisations who work closely with SSWs. The main challenges in providing healthcare to SSWs were services being inflexible, under-resourced services and services not being trauma-informed. Best practice in providing healthcare to SSWs includes- seamless partnership working between agencies with case worker support; peer-involvement in service development and engagement, a range of health provision including outreach, presence in community spaces and fast-track access into mainstream services; trauma-informed, gender-sensitive health services in a welcoming environment with flexible, responsive appointment and drop-in systems and consistent clinicians with specialist knowledge of substance misuse, mental health, domestic violence and homelessness.

**Conclusions:**

Access to healthcare for SSWs in the UK is highly variable but largely inadequate with regards to primary care and mental health provision. The examples of positive healthcare provision and partnership working presented here demonstrate the feasibility of accessible healthcare that meets the needs of SSWs. These need to be systematically implemented and evaluated to understand their impact and implications. As we build back from COVID-19 there is an urgent need to make accessible healthcare provision for marginalised groups the norm, not the exception.

**Supplementary Information:**

The online version contains supplementary material available at 10.1186/s12913-022-07581-7.

## Background

Street sex workers (SSWs) are a highly marginalised and stigmatised group who carry an extremely high burden of unmet health need. They experience multiple and interdependent health and social problems [1] and extreme health and social inequality [2–4]. Sex workers are a heterogenous group; street sex workers carry the highest burden of morbidity [5].

Despite high rates of chronic disease, reproductive health need, respiratory disease and health problems related to substance misuse [2, 5, 6], most clinical services for SSWs (and evaluations) predominantly focus on sexual health [7–9]. Street sex workers have often experienced extensive trauma including child abuse and domestic and sexual violence [2]. They frequently experience poor mental health, particularly anxiety, depression, isolation, post-traumatic stress disorder, self-harm, and suicide [2, 6, 10]. They are frequently excluded from mental health services due to concurrent substance misuse, termed dual diagnosis, which we know to be a common response to adverse experience and extensive trauma [11]. Trauma-informed care is based on understanding and responding to how trauma affects the survivor and is increasingly recommended as an approach in supporting survivors. There is a considerable gap in healthcare services meeting the needs of street sex workers [12, 13] and little evidence for how to fill this gap with holistic, effective care.

In the UK there is geographical variation in sources of healthcare for street sex workers. Author LP delivers a drop-in general practice clinic at a charity for SSWs in Bristol, to our knowledge this level of specialised general practice outreach is only available in one other city in the UK, in Leeds. There are 77 specialist primary health care services in urban areas of England for people who are homeless [14], some SSWs access care through these. In response to local need, some services have adapted or more specialist healthcare providers or pathways have been developed, often in collaboration with charities and advocates, in attempts to better meet the needs of SSWs. Inclusion health is a service, research, and policy agenda that aims to prevent and redress health and social inequities among the most marginalised populations, including people with experiences of homelessness and sex work [15]. There is an absence of high quality evidence for effective healthcare provision for SSWs and this has been highlighted as a priority for further research [15]. Despite the evidence gap, there is considerable front-line experience in people and organisations who have worked with street sex workers often for many years. Our study aims to synthesise the insight and experience of these professionals in the UK.

In this paper we report the perspectives of front-line staff who work with street sex workers in the UK on what healthcare services are available to their clients, how they are provided, how accessible and effective they are, and provide a practical summary of key considerations for providers in designing and developing healthcare for SSWs.

## Methods

Data were collected by a mixed-methods survey (see supplementary material). All methods were performed in accordance with good practice guidance [16]. The survey was administered online using https://www.onlinesurveys.ac.uk/ between April and August 2019.

### Participants

We aimed to characterise perspectives from a range of professionals currently working with street sex workers across the UK. We defined *currently* as within the last year and defined the type of work as *any* capacity. This was purposefully broad as we wanted to capture a range of professional perspectives. Known organisations, experts in the field and networks outlined in Table 1 were contacted by email or Twitter to inform them of the survey and to consider participating if they worked with street sex workers. They were also asked to pass the survey on to other relevant organisations or individuals who work with street sex workers. UK cities which did not have any respondents were then targeted by internet searching using the name of the city and ‘street sex worker’- any relevant organisations found were emailed or telephoned to invite them to participate.

**Table 1:** Networks contacted by email or Twitter to recruit services or individuals who work with street sex workers in the UK

• Sex Work Research Hub https://www.york.ac.uk/spsw/research/swrh/
• The national network of Independent Domestic and Sexual Violence Advocates
• Fair Health UK https://fairhealth.org.uk/
• The Faculty for Homeless and Inclusion Health https://www.pathway.org.uk/faculty/
• London Network of Nurses & Midwives Homelessness Group
• UCL Collaborative Centre for Inclusion Health https://www.ucl.ac.uk/epidemiology-health-care/research/epidemiology-and-public-health/research/ucl-collaborative-centre-inclusion-health
• Members of the AVA Community of Practice on Women’s Multiple Disadvantage

### Survey development

The survey was piloted through two rounds of cognitive interviewing [17], with a convenience sample of three frontline staff from a service that works with street sex workers, in order to evaluate the face validity of the survey. Piloting highlighted problems with question comprehension, interpretation, and response. Following each round of cognitive interviewing, the study team met to discuss the results and necessary refinements were made. The final version of the survey contained 12 multiple choice questions and free text spaces.

The survey asked participants how accessible and how well a service meets the needs of street sex workers in their area. Regarding primary care, these questions were asked about mainstream general practices, homeless health services and any other local primary care outreach services. Respondents were encouraged to elaborate on their answers in free text. These questions were also asked about mental health, sexual health and drug and alcohol services, enquiring separately about mainstream and any other provision, for example outreach services. Participants were then asked what they felt were the main challenges of providing healthcare to street sex workers, any examples of best practice, and what would be important in designing a healthcare service for street sex workers.

### Analysis

Quantitative data were analysed using descriptive statistics including frequencies and percentages. Qualitative responses were imported into Excel, enabling an overview of the data. LP coded the data and generated and analysed themes [18] from the codes which were reviewed by co-authors. The integration of qualitative and quantitative data used the established *following a thread* technique – tracing key themes using all data sets [19]. This included using quotations from themes that provided relevant context or explanation for quantitative findings. Shortlisted quotations were reviewed by a second author JH, who ensured a range of professionals and perspectives were represented, relevant to the provision being explored.

### Patient and public involvement

Staff and women who attend a drop-in that supports street sex workers were consulted on the need for and design of this study. Staff reflected on the variation in healthcare provision in different areas and the reliance on individuals going ‘above and beyond’ to enable care. They were interested to find out what happens in other areas. Women who attended the drop-in reflected that they thought it would be hard to get the participation of street sex workers from different areas, but that asking those who work closely with them was a good idea. The charity supported participant recruitment with their networks and social media.

## Results

The survey received 50 responses from 35 different areas. 43 were from England, five were from Scotland, two from Wales and none from Northern Ireland. Participants were from a range of professionals and the majority of respondents held non-clinical roles (Table 2).

**Table 2:** Participant roles and categorisation

Non-clinical (34)	Number of participants	Category used in quotations^a^
Charity manager/ service coordinator	15	Charity manager (15)
Support/ outreach worker	10	Support worker (10)
Substance use worker	3	Drug and alcohol worker (4)
Charity director	2	Charity director (2)
Police officer	2	Emergency service staff (3)
Commissioner	1	not quoted
Chaplain	1	not quoted
**Clinical (16)**		
Sexual health nurse	4	Sexual health nurse (4)
Sexual health outreach worker	5	Sexual health outreach worker (5)
Clinical Lead/ lead consultant	3	Clinical lead (3)
Non-medical prescriber nurse	1	Drug and alcohol worker (4)
Paramedic	1	Emergency service staff (3)
Practice Nurse	2	Practice nurse (2)

^a^categories used to reduce the risk of individuals being recognised by their role when reporting quotes, the total number in each category is supplied in brackets

### Primary care provision

#### Mainstream primary care

Almost half of respondents felt mainstream primary care was very accessible or mostly accessible to street sex workers in their area, while half felt it was inaccessible or mostly inaccessible (Table 3). The vast majority of respondents felt mainstream primary care did not adequately meet the needs of street sex workers in their area.

**Table 3:**
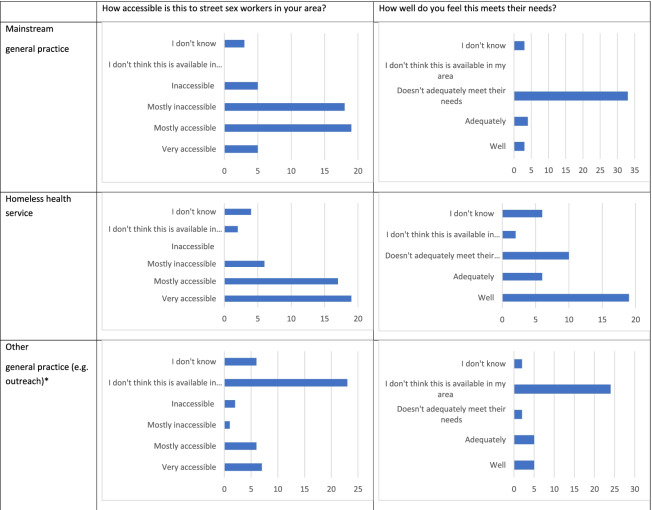
Primary care service provision- how accessible is this to street sex workers and how well does it meet their needs?

^*^4 responses excluded as they were describing a sexual health outreach service

When asked to explain their answer, the most common themes in the responses were that the appointment system was not appropriate to their needs (13 respondents, 26%), street sex workers had difficulty registering with the GP surgery (12 respondents, 24%), difficulty attending the appointment for example because of appointment times (9 respondents, 18%) and fear or experience of stigma or judgment from staff (8 respondents, 16%).

Barriers related to appointment systems included difficulty calling early in the morning for an appointment, not having a phone to be called back on and difficulty attending an appointment booked in advance when their circumstances including housing often change rapidly. Contextual factors were also raised as reasons that mainstream primary care did not meet their needs-

*They struggle with patience and sitting in a GP waiting room with other people can be a stressful experience (Charity manager 8)*

*where women are traumatised or angry or mistrusting, they may end up turned away or even barred… no certainty that access to female medical staff and to trained female professional interpreters available, often have to explain things at front desk overheard by all - a major bar to women in prostitution who feel stigmatised and judged etc (Charity manager 1)*

Some respondents described supplementary support and advocacy to try and overcome these barriers for SSWs, however this was not statutory support and appeared to be happening in only a few areas rather than throughout the UK. The difference between a street sex worker theoretically being able to access primary care and the availability of support/ special arrangements to enable access was apparent:

*Technically accessible and there is some awareness of the barriers to accessing services faced by women in prostitution but patchy implementation of good practice which can facilitate access. (Charity manager 1)*

*I have an arrangement with a GP who is one of the practice partners to fast track SSW’s for access to health care. I am able to accompany them to their appointments. (Drug and alcohol worker 2)*

Of the seven respondents who said that mainstream general practices met the needs of street sex workers in their area ‘adequately’ or ‘well’, three were advocates who supported street sex workers to be able to access the service and two were clinicians themselves.

#### Homeless health primary care services

As shown in Table 3, where specialist homeless health surgeries exist and are known about, the vast majority of respondents rated them as mostly or very accessible. Where respondents felt able to comment on how well these services met their needs, they judged them better than mainstream primary care: almost three quarters of respondents who gave an opinion on this said that the service met their needs ‘adequately’ or ‘well’, with most reporting ‘well’.

In free text answers many respondents (16 out of 35, 45.7%) commented on some aspects of homeless health services providing a better model of care for street sex workers, including appointment flexibility, expertise of staff, access to multiple aspects of support at one site or the ability to ‘drop in’ to seek care. However, respondents also highlighted inconsistency in availability and the challenge of the service being in high demand.

*Homeless Health has the right model to meet the needs of sex workers. Health interventions are mainly all in one place and this works for the women. (Charity manager 2)*

*We have a good specialised homeless GP practice in [location] where staff are generally understanding to the clients needs, including the need for flexibility and last minute appointments. There have been some inconsistencies amongst staff when requesting pre-bookable appointments but generally willingness and availability is good. (Support worker 9)*

*These services are better train and equipped to worker with SSW, however demand is too high and therefore are unable to meet the needs of those most vulnerable. (Charity manager 4)*

While most respondents perceived homeless health services as a better model for street sex workers than mainstream primary care, some highlighted that street sex workers may not be homeless which could be a barrier to them accessing care, as well services not always easily accessible to women.

*the waiting room is not always a safe place for women to be because of the presence of dealers or perpetrators. (Support worker 2)*

*the location of [homeless health surgery] is based near a very male dominated hostel, which acts as a barrier to the women, from fear of provocation, and previous histories and general fear of the men/males whom frequent [the hostel]. (Charity manager 9)*

*women find it difficult to access treatment outside women only services (Drug and alcohol worker 4)*

One respondent highlighted the difficulties of transitioning patients to mainstream care.

*[drug and alcohol charity] workers also play a role is supporting patients to access mainstream services once they have accommodation. This can be challenging as many women have poor experiences of mainstream services and want to stay with [drug and alcohol charity] who are more flexible and understanding of their needs” (Drug and alcohol worker 3)*

#### Other outreach primary care services

Some areas have a primary care outreach service which may be more targeted to street sex workers. As shown in Table 3, the majority of respondents reported they were not aware of this in their area. Free text responses identified eight different providers of primary care outreach in the UK for street sex workers that respondents were aware of; two of these were a nurse-led service.

There were five respondents who rated the outreach primary care service as ‘very accessible’ and felt they met their needs ‘well’; four of these were with regards to a GP who attended a drop-in centre, and the other was for a fast-track arrangement with a local general practice.

Where there was some kind of primary care outreach, this was praised by respondents, but often relied on key dedicated staff and capacity limitations were noted:

*the GPs at [organisation] go above and beyond to see as many women is as physically possible, overstaying their hours to treat the women. We totally value and respect the GP's commitment to the women whom they support and treat. (Charity manager 9)*

*We have some amazing local support from a named GP who has really advocated on behalf of the workers and strives to ensure access to healthcare for them, but it feels that this is more individually led than organisationally driven. (Emergency service staff 2)*

*Practice nurse attends sex worker outreach services through [organisation] and another local agency but this is only for basic care and referrals. (Charity director 1)*

A further four outreach services were described as a response to the question about GP outreach but looking at the detail it was clear these respondents were actually describing sexual health outreach.

Where primary care outreach was available, opinion was spread as to whether this provision adequately met their needs. The most frequent response was ‘don’t know’, followed by ‘well’.

Despite not being asked this, one respondent raised a willingness to collaborate with primary care and support outreach GP services for street sex workers.

*[Organisation] would be very happy to partner with an outreach GP to improve services for our clients. (Charity manager 3)*

### Other health service provision

#### Mainstream mental Health

Three quarters of respondents felt mainstream mental health services were inaccessible or mostly inaccessible to SSW and did not meet their needs (Table 4). Experience of trauma, fear of stigma and difficulty trusting mainstream services were raised as access barriers, along with difficulties in the referral and assessment processes.

*women who have experienced trauma, who had multiple ACE's (adverse childhood experiences), who feels stigmatised or have addiction problems often report having no trust in mainstream services. (Sexual health nurse 4)*

**Table 4:** Other health service provision- how accessible is this to street sex workers and how well does it meet their needs?

	Mainstream mental health service	Other mental health service (e.g. outreach)	Mainstream sexual health service	Other sexual health service (e.g. outreach)	Mainstream drug and alcohol service	Other drug and alcohol service (e.g. outreach)
How accessible is this to street sex workers in your area?	*n* = 50	*n* = 50	*n* = 50	*n* = 50	*n* = 50	*n* = 45
Very accessible	0 (0.0%)	1 (2.0%)	16 (32.0%)	21 (42.0%)	12 (24.0%)	11 (24.4%)
Mostly accessible	10 (20.0%)	14 (28.0%)	17 (34.0%)	11 (22.0%)	26 (52.0%)	16 (35.5%)
Mostly inaccessible	25 (50.0%)	11 (22.0%)	15 (30.0%)	5 (10.0%)	9 (18.0%)	6 (13.3%)
Inaccessible	11 (22.0%)	3 (6.0%)	1 (2.0%)	1 (2.0%)	1 (2.0%)	0 (0.0%)
I don't think this is available in my area	1 (2.0%)	12 (24.0%)	0 (0.0%)	7 (14.0%)	0 (0.0%)	8 (17.8%)
I don't know	3 (6.0%)	9 (18.0%)	1 (2.0%)	5 (10.0%)	2 (4.0%)	4 (8.8%)
How well do you feel this meets their needs?	*n* = 44	*n* = 40	*n* = 44	*n* = 41	*n* = 44	*n* = 36
Well	1 (2.3%)	4 (10%)	12 (27.3%)	18 (43.9%)	11 (25.0%)	10 (27.8%)
Adequately	5 (11.4%)	6 (15.0%)	13 (29.5%)	9 (22.0%)	16 (36.4%)	9 (25.0%)
Doesn't adequately meet their needs	34 (77.3%)	13 (32.5%)	18 (40.9%)	6 (14.6%)	15 (34.1%)	9 (25.0%)
I don't think this is available in my area	1 (2.3%)	12 (30.0%)	0 (0.0%)	6 (14.6%)	2 (4.5%)	3 (8.3%)
Don't know	3 (6.8%)	5 (12.5%)	1 (2.3%)	2 (4.9%)	0 (0.0%)	5 (13.9%)

Difficulties in the referral and assessment processes were highlighted as barriers to street sex workers accessing mental health care.

*Having to attend the GP to get a referral, wait for a long time… for SSWs, the DNA [did not attend] procedure can be particularly difficult as they may need to be re-referred several times. (Support worker 6)*

*Mainly the referral and assessment process is difficult to engage in - the women I work with won't answer the phone to a withheld or unknown number, regularly change their phone numbers, often don't open post. (Charity manager 8)*

Dual diagnosis (co-occurring mental illness and substance misuse) was raised by several respondents as a barrier to accessing mental health services; only one respondent mentioned access to a substance use psychiatrist.

*Mental health services are entirely inadequate for women facing multiple disadvantage. Women who are using substances are told that they cannot access mental health support until they've addressed their problematic substance use. Women who are in mental health crisis are told they need to attend drug treatment and given no support from mental health services. (Support worker 2)*

#### Outreach mental health

The majority of participants thought outreach mental health services in their area were *mostly accessible* or did not know if they were available. The most frequent free text comments were that outreach mental health services were unavailable or that there was some outreach but it was under-resourced. Two respondents described provision that was available only if women commit crime; one reported they had good access because an outreach mental health worker was connected to their team.

#### Sexual health and drug and alcohol services

The majority of participants thought sexual health and drug and alcohol services were accessible and responsive to SSWs. Participants described flexibility of the service and the involvement of an advocate as enabling access to care.

*We've found our local sexual health service to be very flexible and accommodating. We have an arrangement where we can present a woman to the clinic and she is automatically placed at the front of the queue. (Support worker 2)*

*Drug and alcohol services are generally very accessible if the client is supported through another organisation. For example, we have fast track access through named sex work lead workers at each branch of the drugs service. (Support worker 9)*

The impact of flexible arrangements on engagement in substance misuse treatment in different areas was stark between services:

*Referral queue system and penalties for missed appointments mean that there are very few women we work with on scripts or in any form of treatment. (Support worker 7)*

*We have brilliant drug and alcohol services and the overwhelming majority of our working women are scripted. (Emergency service staff 2)*

Sexual health outreach was the highest rated of the outreach services for both accessibility and meeting needs. The majority described services as a collaboration between sexual health services and charities who support SSWs.

*The sexual health worker in the city joins us on our evening outreach once a month. She also has access to fast appointments for contraception and will do sexual health checks whenever we take a woman to see her or come out to us. (Charity manager 10).*

There were similar collaborations described with drug and alcohol workers joining support services on outreach that were well regarded. Gender insensitivity was raised as a barrier to reaching SSWs:

*Outreach is in hostels and at day centres rather than on-street. Not all of our women access these services so are missed. The day centres are mixed so don't provide a safe women-only space. (Support worker 2)*

### Main challenges in providing healthcare to street sex workers

When asked ‘What do you think the main challenges are in providing effective healthcare to street sex workers?’ most responses described problems with services or staff, rather than the street sex workers themselves. The most common theme described (20/ 50) was the lack of flexibility of services. Other frequently raised themes related to the services included that they were not trauma-informed and were under-resourced.

*Mainstream services are too regimented for street sex workers to engage with, the approach needs to be flexible and responsive, appointments and penalising for non-engagement creates barriers. (Charity manager 11)*

*Zero tolerance policies in health services; women with complex trauma being labelled aggressive and barred from services (Support worker 6)*

Responses that discussed healthcare staff highlighted knowledge, experience and attitudes of professionals as potential challenges in providing effective healthcare to street sex workers:

*Not having access to people with an understanding of the complexity of SSWs lives who will look at more than one health problem at a time. (Charity manager 10)*

*Multiple complex needs - e.g. homelessness, mental health, and addiction combined, make it hard to get appropriate health care and for some health professionals to understand the many barriers in place. (Charity Director 1)*

Trust was raised as a key challenge, and respondents highlighted relationships and consistency as necessary enablers of this:

*Trust - taking services to women rather than women coming to services helps but generally have to build a sustained trusting relationship before effective and sustained engagement can follow through (Charity manager 1)*

*Building trust and rapport takes time, engagement is unpredictable, need to work flexibly and opportunistically.* (Charity manager 6)

### Best practice in providing healthcare to street sex workers

Participants were asked about examples of best practice, and what is most important in the design of a healthcare service for street sex workers. Answers provided were the most detailed and thorough responses in the whole survey; respondents were enthusiastic to share their opinions. Best practice in providing healthcare to SSWs includes- seamless partnership working between agencies with case worker support; peer-involvement in service development and engagement, a range of health provision including outreach, presence in community spaces and fast-track access into mainstream services; trauma-informed, gender-sensitive health services in a welcoming environment with flexible, responsive appointment and drop-in systems and consistent clinicians with specialist knowledge of substance misuse, mental health, domestic violence and homelessness. Table 5 summarises key points for providers to consider in designing and developing healthcare provision for SSWs, alongside valuable context and explanation from frontline workers which informs these key points.

**Table 5:** Best practice guidance from participants

Summary key points for providers	Example quotes from frontline workers
**Partnership working and a range of provision:** • Outreach • Provision in community spaces • Network of agencies to provide holistic support • Fast track appointments • Seamless pathways between agencies • Good communication between agencies	*• the ideal would be for services to visit where sex workers feel most comfortable and then for assessment, referral and appointment booking all to be done in one hit. This would acknowledge how rare chances are that people feel ready to engage and would maximise the potential in those rare instances. (Support worker 7)* *• Providing an in-house nurse/healthcare worker available specifically for sex workers- reduces stigma and judgements towards sex workers often arising from other services. (Support worker 5)* *• In house GP presence in drop-in who have a good understanding and awareness of the needs and risks the women face. GP's link with other services to provide joined up approach. (Charity manager 13)* *• offer of immediate care *via* quick interventions (eg on street medicine), take healthcare to them—some individuals will never / rarely access healthcare in a professional setting (Support worker 9)* *• We work extensively with mental health colleagues, health inclusion nurses, addiction key workers, safeguarding midwives, voluntary sector to identify women with unmet needs and then work creatively and flexibly to meet them out in the community where they are (Charity manager 6)* *• Strong relationships between key individuals in relevant agencies… means that despite system not being ideal, there is a good network of support for sex workers (Charity director 1)* *• [Charity] run a service for sex workers and are willing to work jointly with Drug and Alcohol services—this can build up trust and encourage engagement (Drug and alcohol worker 1)* *• Strong communication between different systems to avoid repetition of information (Charity director 1)*
**Organisation of health services:** • Drop-in availability • Afternoon and out of hours provision • Longer appointments • Psychologically informed environment • Flexible and responsive	*• There are several afternoon sessions that people can access (Support worker 6)* *• Easy access to GP support and prescriptions out of hours—most women we work with struggle during normal working hours to access support. (Charity director 2)* *• Time to address several health needs at once. (Charity manager 10)* *• Ensure the service was reactive, provide healthcare/ support when the person was ready not at an appointment 3 weeks away. (Charity manager 11)* *• The service would need to be designed to allow for multiple consultations where there is no 'concrete' outcome—that the outcome is building trust and rapport. Commissioners need to understand that partial outcomes for our patients is progress. (Charity manager 6)* *• A really nice environment where women would like to come and be. If they do have to wait, can the waiting room be lovely? Can there be things to do whilst they wait? Can they chat to a housing worker whilst they wait? A nice cup of tea? (Support worker 2)* *• a more relaxed accessible drop-in to access treatment for dressings legs *etc*. At these appointments she has often requested a doctor to come and see them at the same time and refer them for other health needs whilst we have them there. (Charity manager 10)* *• Our service does not discharge people for non-attendance. We will actively and creatively think of ways to engage. (Clinical lead 1)*
**Health professionals and staff- expertise and approach:** • Trauma-informed • Specialist knowledge of substance misuse, mental health, domestic violence, homelessness • Trusted relationships • Non-judgemental • Continuity of care • Psychological support for staff	*• they provide really good follow up, they link in with other services who work with the person, they have good awareness of how sexual trauma affects women when accessing something like a smear test (Support worker 6)* *• Health care professionals understanding impact of complex trauma, not being dismissive of a SSW due to substance use and taking the time to let the individual be heard around their health. (Charity manager 4)* *• Ability to address/treat mental health alongside substance misuse. (Charity manager 13)* *• providing the right training to staff (trauma informed practice, borderline personalities training, a good understanding of the impact of ACE's and a good knowledge of health and gender inequalities. (Sexual health nurse 4)* *• when these vulnerable women do access a service they are seen by a professional who is trained to support as fully as possible without having to go to another appointment/service. (Charity manager 6)* *• Consistency of staff/staff who believe in what they are doing and understand the specific needs of SWs (Sexual health nurse 3)* *• Really good mental health practitioners who are specifically trained to support this client group and who care about them. (Support worker 2)* *• flexibility, non-judgmental healthcare professionals (no stigma!), confidence in confidentiality, compassion (Charity manager 10)* *• Sex workers do not appreciate many different people as they find it difficult to trust and difficult to explain things multiple times. (Sexual health outreach worker 3)* *• Psychological support for staff for reflective practice to consider transference and parallel process. (Clinical lead 1)*
**Case worker support:** • Holistic • Advocacy • Creative ways to engage and support	*• assertive outreach that helps women to stay with treatment and advocates for women and accompanies them in their journeys. (Charity manager 1)* *• We work in a person-centred way, supporting women 'where they are at' to achieve the goals that they have in mind. Often this leads to more in-depth interventions, support to access health care and substance use support. (Drug and alcohol worker 3)*
**Peer and volunteer involvement**	*• Peer mentor and volunteer support at [drug service] appears helpful for engagement (drug and alcohol worker 1)* *• Many projects fail in this area because they do not involve sex workers in the planning and set up of any services. (Sexual health nurse 1)* *• Peer led works really well (Practice nurse 2)*
**Gender sensitivity**	*• It would have workers well trained/experienced in the specific needs of sex workers (including trans sex workers) who are non-judgemental (Support worker 6)* *• Female only settings (Charity manager 13)*

## Discussion

In the context of scant evidence of effective healthcare for SSWs, this paper presents the perspectives of a range of UK front-line professionals who work with SSWs on the accessibility of healthcare services currently available to their clients, whether it meets their needs and their views on how to best provide healthcare to this group.

### Mainstream healthcare

Mainstream general practice was not meeting the needs of SSWs unless there was a fast-track arrangement or advocacy support in place, which was rare. Mainstream mental health provision was largely inaccessible and irresponsive to dual diagnosis, despite the existing evidence base and guidelines [20]. The benefits of the more flexible and inclusive approaches of sexual health and drug and alcohol services described in this study were clear. It is also clear that services at intense financial pressure and at risk of overwhelm struggle to maintain access and flexibility [21, 22].

### Homeless Health

Where homeless health services were available, they were more accessible and met the needs of SSWs better than mainstream provision. This is reflected by the difficulty and reluctance patients can find in transitioning to mainstream care when they are no longer homeless, often requiring additional support [23]. Concerns raised in this study about demand and safety of homeless health services are critical factors to consider in designing services for people who have experienced extensive trauma [24], and are detailed in good practice guidance [25]. While there are often shared experiences between the homeless population and street sex workers, there are also important differences, and the needs of both groups need to be considered in inclusion health. Gender-sensitivity is an important consideration in this, however most homeless health provision does not have women-only provision [14].

### Outreach

This study found very few examples of primary care and mental health outreach nationally. National standards recommend specialist outreach services for sex workers as a vital way to improve access and should include enhanced access to primary care and not be confined to sexual health and contraception [26]. Four respondents described a sexual health outreach service when asked about primary care outreach- highlighting the inaccurate perception that the health needs of SSWs are limited to sexual health, even amongst those who work closely with them.

### Barriers and enablers

Comparing these results to a survey of 71 SSWs in a UK city reveals considerable overlap in describing the main barriers as “appointments, waiting times, and fear of judgement and other patients staring” and a suggested strategy of collaborative provision of an “integrated service providing basic living needs alongside health care” [2]. Barriers and enablers described in this study echo the literature on trauma-informed care [24], whereby both the physical arrangement of the service and the attitudes of staff can re-traumatise those who have already experienced extensive trauma, potentially deterring the service-user from further engagement.

A strength of this study is the professional diversity of participants represented, reflecting the breadth of professionals that can be involved in the support of SSWs. This study captured a range of perspectives on current provision as well as coherent, practical themes on how to improve healthcare for SSWs. The perspectives of those who work closely with SSWs may be different to hearing directly from SSWs themselves and the findings should be interpreted considering this limitation.

Despite our efforts, we had few responses from Scotland and Wales and no responses from Northern Ireland. Findings should be interpreted in light of this limitation. Northern Ireland has a more criminalised legal model of sex work than the rest of the UK, which we know to be more harmful to health [9], and may reduce the applicability of our findings here.

This survey was conducted prior to the COVID-19 pandemic, which has widened inequalities and decreased access to healthcare for marginalised groups [27]. The full impact of COVID-19 on SSWs is not yet fully understood, but it is likely that the imperative to improve access to trauma-informed healthcare for marginalised groups has only become more important and urgent.

## Conclusions

Failing to provide accessible healthcare to SSWs harms individuals, families and health services. Effective primary care, mental health, sexual health and drug and alcohol services are all needed in this population; it is evident that access to healthcare that meets the needs of SSWs in the UK is variable but largely inadequate, particularly with regards to primary care and mental health. The examples of positive healthcare provision and partnership working presented here demonstrate the feasibility of accessible healthcare that meets the needs of street sex workers. We have articulated best practice guidance that needs implementation and further evaluation. As we build back from COVID-19 there is an urgent need to make accessible, effective healthcare provision for marginalised groups the norm, not the exception.

## Supplementary Information

**Additional file 1.**

## Data Availability

All data generated or analysed during this study are available on request at the University of Bristol data repository, data.bris, at https://doi.org/10.5523/bris.35j0s1qxm21bf2fv8xyptxoncj.

## Abbreviations

GP: General Practitioner (Family Doctor)
SSW: Street sex worker
UK: United Kingdom
