# Supplementary material for: Access to healthcare for street sex workers in the UK: perspectives and best practice guidance from a national cross-sectional survey of frontline workers
Source: BMC Health Serv Res. 2022 Feb 11;22:178. doi: 10.1186/s12913-022-07581-7 (PMC8840502; doi:10.1186/s12913-022-07581-7)
Supplement: Supplementary file 1 — Additional file 1. [file 12913_2022_7581_MOESM1_ESM.pdf]

# Healthcare for street sex workers

## About this survey

**This is a national survey about whether adult street sex workers have access to health services that meet their needs. Your insight can help build on good practice and show where things need to change. If you work with street sex workers, in any capacity, please take part.**

This survey is about access to primary health care (meaning healthcare received in the community)- including GP services, mental health, sexual health and drug and alcohol services.

**What are the possible benefits and disadvantages of taking part?**

Although there is no direct benefit to you, by taking part you will have a say in how to improve health services for street sex workers.

**Do I have to take part?**

No, taking part is voluntary and you can stop at any time.

**How will the results of the study be used?**

All information which is collected will be kept strictly confidential. Anonymous data from this survey will be published to help improve health services for street sex workers. No names or identifying details will be reported.

**Who is organising the study?**

The study is organised by the University of Bristol.

**GDPR statement**

As a university we use personally-identifiable information to conduct research to improve health, care and services. As a publicly-funded organisation, we have to ensure that it is in the public interest when we use personally-identifiable information from people who have agreed to take part in research. This means that when you agree to take part in a research study, we will use your data in the ways needed to conduct and analyse the research study. Your rights to access, change or move your information are limited, as we need to manage your information in specific ways in order for the research to be reliable and accurate. If you withdraw from the study, we will keep the information about you that we have already obtained. To safeguard your rights, we will use the minimum personally-identifiable information possible.

Health and care research should serve the public interest, which means that we have to demonstrate that our research serves the interests of society as a whole. We do this by following the UK Policy Framework for Health and Social Care Research.

If you wish to raise a complaint on how we have handled your personal data, you can contact our Data Protection Officer who will investigate the matter. If you are not satisfied with our response or believe we are processing your personal data in a way that is not lawful you can complain to the Information Commissioner's Office (ICO).

If there are any specific comments relating to data protetion, you can contact our Data Protection Officer on: data-protection@bristol.ac.uk.

If you have any questions about completing this survey, please feel free to contact the researcher on the details below. If you would like to an independent contact for complaints, please email research-governance@bristol.ac.uk.

Thank you for your time.

Dr Lucy Potter

Academic GP, Bristol Medical School

Lucy.potter@bristol.ac.uk

## Consent to participate

I agree to my survey answers being used for research ✱ *Required*

- Yes
- No

I consent to (anonymised and unidentifiable) data from my survey answers being stored in the University of Bristol Research Data Repository. More information on this can be accessed at https://www.bristol.ac.uk/staff/researchers/data/accessing-research-data/ (you can still participate in the research even if you answer ‘no’ to this) ✱ *Required*

- Yes
- No

## About your experience and role

Do you have experience of working with street sex workers (in any capacity) ✱ *Required*

- Yes
- No

What is the name of your service/ organisation? ✱ *Required*

Geographical area covered ✱ *Required*

Description of service or link to website ✱ *Required*

What is your role? ✱ *Required*

## Access to GP services

We would like to understand if and how street sex workers (SSWs) have access to **GP services** in your area. Please rate the access you are aware of in your area.

| | How accessible is this service to SSWs in your area? | Please explain | How well do you feel this meets th | | | |
|---|---|---|---|---|---|---|
| | | | Well | Adequately | Doesn't adequately meet their needs | Do kn |
| Mainstream GP surgery | Please select | | | | | |
| Homeless Health GP surgery | Please select | | | | | |
| Other GP service (e.g. outreach) | Please select | | | | | |

If there is an outreach GP service in your area please describe this and if it is specifically for street sex workers

## Access to mental health services

We would like to understand if and how street sex workers (SSWs) have access to **mental health services** in your area. Please rate the access you are aware of in your area.

| | How accessible is this service to SSWs in your area? | Please explain | How well do you feel this meets their needs? | | | |
|---|---|---|---|---|---|---|
| | | | Well | Adequately | Doesn't adequately meet their needs | Do kn |
| Mainstream mental health service | Please select | | ○ | ○ | ○ | ○ |
| Other mental health service (e.g. outreach) | Please select | | ○ | ○ | ○ | ○ |

If there is an outreach/ other mental health service in your area please describe this and if it is specifically for street sex workers

## Access to sexual health services

We would like to understand if and how street sex workers (SSWs) have access to **sexual health services** in your area. Please rate the access you are aware of in your area.

| | | | How well do you feel this meets their needs? | | | |
|---|---|---|---|---|---|---|
| | How accessible is this service to SSWs in your area? | Please explain | Well | Adequately | Doesn't adequately meet their needs | Do kn |
| Mainstream sexual health service | Please select | | ○ | ○ | ○ | ○ |
| Other sexual health service (e.g. outreach) | Please select | | ○ | ○ | ○ | ○ |

If there is an outreach/ other sexual health service in your area please describe this and if it is specifically for street sex workers

# Access to drug and alcohol services

We would like to understand if and how street sex workers (SSWs) have access to **drug and alcohol services** in your area. Please rate the access you are aware of in your area.

| | | | How well do you feel this meets th alcohol care needs? | | | |
|---|---|---|---|---|---|---|
| | How accessible is this service to SSWs in your area? | Please explain | Well | Adequately | Doesn't adequately meet their needs | Do kn |
| Mainstream drug and alcohol service | Please select | | ○ | ○ | ○ | ○ |
| Other drug and alcohol service (e.g. outreach) | Please select | | ○ | ○ | ○ | ○ |

If there is an outreach/ other drug and alcohol service in your area please describe this and if it is specifically for street sex workers

## Your service provision

Does your service/ organisation provide healthcare to street sex workers? ✱ *Required*

- Yes
- No
- Don't know

## Healthcare provision for street sex workers at your service

Please describe the healthcare service that is available ✱ *Required*

## Challenges and areas for improvement

What do you think the main challenges are in providing effective healthcare to street sex workers? ✱ *Required*

Can you share any examples of best practice you have come across in providing healthcare to street sex workers? ✱ *Required*

If you could design a service to best meet the health needs of street sex workers what would be important? ✱ *Required*

## Follow up

If you would be happy to be contacted to follow up on any of your answers, please provide your email address. It will be held securely and will not be passed on to any third parties. You can withdraw your consent at any time.

## Thank you

Thank you for your time.

If you would like to be sent a copy of the final paper of this study, please email lucy.potter@bristol.ac.uk

---

## Key for selection options

**4.1.a - How accessible is this service to SSWs in your area?**
- Very accessible
- Mostly accessible
- Mostly inaccessible
- Inaccessible
- I don't know
- I don't think this is a service available in my area

**4.2.a - How accessible is this service to SSWs in your area?**
- Very accessible
- Mostly accessible
- Mostly inaccessible
- Inaccessible
- I don't know
- I don't think this is a service available in my area

**4.3.a - How accessible is this service to SSWs in your area?**
- Very accessible
- Mostly accessible
- Mostly inaccessible
- Inaccessible
- I don't know
- I don't think this is a service available in my area

**5.1.a - How accessible is this service to SSWs in your area?**
- Very accessible
- Mostly accessible
- Mostly inaccessible
- Inaccessible
- Don't know
- I don't think this is a service available in my area

**5.2.a - How accessible is this service to SSWs in your area?**
- Very accessible
- Mostly accessible
- Mostly inaccessible
- Inaccessible
- Don't know
- I don't think this is a service available in my area

**6.1.a - How accessible is this service to SSWs in your area?**
- Very accessible
- Mostly accessible
- Mostly inaccessible
- Inaccessible
- I don't know
- I don't think this is a service available in my area

**6.2.a - How accessible is this service to SSWs in your area?**
- Very accessible
- Mostly accessible

Mostly inaccessible
Inaccessible
I don't know
I don't think this is a service available in my area

**7.1.a - How accessible is this service to SSWs in your area?**
Very accessible
Mostly accessible
Mostly inaccessible
Inaccessible
Don't know
I don't think this is a service available in my area

**7.2.a - How accessible is this service to SSWs in your area?**
Very accessible
Mostly accessible
Mostly inaccessible
Inaccessible
Don't know
I don't think this is a service available in my area
